# On the Influence of Marshy Exhalations on Phthisis

**Published:** 1844-10-05

**Authors:** James Bryan

**Affiliations:** Philadelphia


### ON THE INFLUENCE OF MARSHY EXHALATIONS ON PHTHISIS.

BY JAMES BRYAN, M. D., OF PHILADELPHIA.

In "Braithwaite's Retrospect" for July, 1844, a quotation from the Gazette Medicale, of Nov., 1843, occurs, in which it is stated that a letter addressed to the President of the Academy of Medicine, on the infrequency of phthisis in marshy districts, was read. Messrs. Oliver, Brera and others, have called the attention of the profession to the fact, that there exists an antagonism between phthisis and marsh fevers. A countryman of our own, Dr. H. Green, of New York, published a very interesting paper some years ago, containing several marked cases confirmatory of this view, in the New York Journal of Medicine and Surgery. A personal acquaintance with Dr. Green, and some knowledge of the localities spoken of, induce me to place great confidence in his statements.

A case, among others, which came under my own notice, gives, in my opinion, additional strength to the testimony. A young lady of delicate conformation and phthisical habit, had laboured for some two years under the usual symptoms of incipient consumption. These, from sedentary habits and other causes, became confirmed, and she left the city unable to walk, or sit up any length of time. She went to a relative in New Jersey, who resides on the margin of a marshy stream, whose farm in fact, embraces a large amount of low ground, fit only for hay and grass. On being removed there, which was only that she might die among her friends, a physician was called in for form sake, without any hope of relief. Contrary to all expectations, at the end of a few months, her night-sweats, expectoration, cough, &c., were much relieved, and a slight "show" of the menstrual discharge, which had entirely ceased for several months, took place. At this time she is able to visit New York, and travels over the country among her friends, apparently in a fair way of recovery.

Other cases might be mentioned but space will not permit. It has occurred to the writer, some of whose family and patients have been relieved by a temporary residence in marshy districts, that if "these things be so," whether, as medical advisers, we might not send our patients (during the spring and autumn months) to reside on the banks of the Schuylkill among the "haunted houses" north and west of the city, or to the low grounds of Delaware, Maryland or New Jersey. This would certainly be more convenient than sending them to distant and foreign countries, exposed to the many privations inseparably connected with long journeys. Will not medical men, who reside in miasmatic districts, give their experience in these matters, and inform us of the frequency of pulmonary diseases where malaria gives the prevalent endemic type?

